# Lipidomics Reveals Early Metabolic Changes in Subjects with Schizophrenia: Effects of Atypical Antipsychotics

**DOI:** 10.1371/journal.pone.0068717

**Published:** 2013-07-24

**Authors:** Joseph McEvoy, Rebecca A. Baillie, Hongjie Zhu, Peter Buckley, Matcheri S. Keshavan, Henry A. Nasrallah, George G. Dougherty, Jeffrey K. Yao, Rima Kaddurah-Daouk

**Affiliations:** 1 Duke University Medical Center, Department of Psychiatry and Behavioral Sciences, Durham, North Carolina, United States of America; 2 Rosa & Co LLC, San Carlos, California, United States of America; 3 Medical College of Georgia, Augusta, Georgia, United States of America; 4 Beth Israel Deaconess Medical Center and Harvard Medical School, Boston, Massachusetts, United States of America; 5 University of Cincinnati College of Medicine, Cincinnati, Ohio, United States of America; 6 VA Pittsburgh Healthcare System, Pittsburgh, Pennsylvania, United States of America; 7 Department of Psychiatry, University of Pittsburgh School of Medicine, Pittsburgh, Pennsylvania, United States of America; Rikagaku Kenkyūsho Brain Science Institute, Japan

## Abstract

There is a critical need for mapping early metabolic changes in schizophrenia to capture failures in regulation of biochemical pathways and networks. This information could provide valuable insights about disease mechanisms, trajectory of disease progression, and diagnostic biomarkers. We used a lipidomics platform to measure individual lipid species in 20 drug-naïve patients with a first episode of schizophrenia (FE group), 20 patients with chronic schizophrenia that had not adhered to prescribed medications (RE group), and 29 race-matched control subjects without schizophrenia. Lipid metabolic profiles were evaluated and compared between study groups and within groups before and after treatment with atypical antipsychotics, risperidone and aripiprazole. Finally, we mapped lipid profiles to n3 and n6 fatty acid synthesis pathways to elucidate which enzymes might be affected by disease and treatment. Compared to controls, the FE group showed significant down-regulation of several n3 polyunsaturated fatty acids (PUFAs), including 20:5n3, 22:5n3, and 22:6n3 within the phosphatidylcholine and phosphatidylethanolamine lipid classes. Differences between FE and controls were only observed in the n3 class PUFAs; no differences where noted in n6 class PUFAs. The RE group was not significantly different from controls, although some compositional differences within PUFAs were noted. Drug treatment was able to correct the aberrant PUFA levels noted in FE patients, but changes in re patients were not corrective. Treatment caused increases in both n3 and n6 class lipids. These results supported the hypothesis that phospholipid n3 fatty acid deficits are present early in the course of schizophrenia and tend not to persist throughout its course. These changes in lipid metabolism could indicate a metabolic vulnerability in patients with schizophrenia that occurs early in development of the disease.

## Introduction

Schizophrenia is a serious psychiatric brain disorder with a worldwide prevalence of about 1% [1]. Due to its early age of onset, schizophrenia has a large socioeconomic impact, with long-term disability and associated caretaking costs [2]. Previous work had implicated neurotransmitters and receptors in the pathophysiology of schizophrenia; thus, most therapeutic drug development has targeted dopamine, serotonin, and glutamate systems. Antipsychotics, which work by blocking dopamine, are highly effective in treating psychosis, but the vast majority of individuals discontinue treatment over time, due to lack of effectiveness or development of side effects; furthermore, not all patients respond similarly to those medications [3].

Schizophrenia is linked to a high prevalence of comorbid medical disorders. For example, diabetes is 2 to 4 times more prevalent in patients with schizophrenia than in the general population [4,5]. Many medical disorders, including metabolic syndrome, obesity, diabetes, and cardiovascular disease, are characterized by changes in lipid metabolism. Moreover, several antipsychotic drugs, particularly those that target dopamine and serotonin receptors (e.g., clozapine and olanzapine), can cause adverse effects that are related to lipid metabolism, including weight gain, insulin resistance, and hypertriglyceridemia [6]. However, other drugs have reduced side effects; for example, a 26-week randomized clinical trial tested 317 patients with schizophrenia and found that aripiprazole (ARI) was associated with less weight gain and smaller increases in total cholesterol/triacylglycerol than olanzapine [7].

It is not clear whether patients with schizophrenia are predisposed to metabolic disorders (i.e., before treatment) or whether these disorders are primarily a treatment effect. Accordingly, several studies have focused on patients with first episodes of schizophrenia to investigate whether patients with no drug exposure were more likely to have developed a metabolic disorder than controls. However, those studies showed inconsistent results [8,9].

In recent decades, several studies have shown that phospholipids were impaired in schizophrenia [10,11]. Phospholipids play a critical role in the structure and function of membranes (including synaptic vesicle membranes); therefore, changes in membrane lipids might directly affect neurotransmission [12,13]. In a previous study, we found evidence that atypical antipsychotics had peripheral lipid effects [11]. We found that schizophrenia was associated with major defects in polyunsaturated fatty acid (PUFA) compositions in phosphatidylethanolamine (PE) and phosphatidylcholine (PC) lipid classes. In addition, PE synthesis was reduced in schizophrenia. We also found that treatment with antipsychotic drugs partially reversed the deficits in PE concentrations observed in patients with schizophrenia. Moreover, different drugs affected different lipids; for example, olanzapine and risperidone (RIS) affected a much broader range (~50 lipids) than ARI.

The most functionally significant lipids are PUFAs, which comprise two main classes: those with the first double bond at the 3^rd^ carbon atom (n3 PUFAs) and those with the first double bond at the 6^th^ (n6 PUFAs) carbon atom, counting from the methyl end of the fatty acid chain. Both n3 and n6 fatty acids are abundantly found in the brain [14]. In particular, arachidonic acid (n6), dihomogamma-linolenic acid (n6), and eicosapentaenoic acid (EPA, n3) are important in cell-signaling and enzyme-regulation, and they serve as precursors for eicosanoids (prostaglandins, thromboxanes, and leukotrienes). The n6 fatty acids, like arachidonic acid, tend to promote inflammatory conditions [15]. In contrast, the n3 fatty acids tend to reduce the rate of inflammatory molecule production.

The first aim of the present study was to investigate which lipid changes were associated with schizophrenia, early in the disease, and which developed after taking antipsychotic drugs, later in the disease. A second aim was to determine where in the lipid synthesis pathway these effects occurred. We examined lipid profiles derived from the lipidomics platform. Then, we examined the changes in lipids that occurred after 2 weeks of antipsychotic treatment (RIS or ARI) for schizophrenia.

## Methods

This study was approved by the Duke University Institutional Review Board. Written, informed consent was obtained from all subjects or legal guardians. Clinicians were blinded to the laboratory data, and laboratory assessments were carried out blinded to diagnostic assignments of the subjects.

### Selection criteria

#### Patients

The subjects presented here have been described previously [16]. Briefly, 20 subjects were medication-naïve, and they had presented for treatment at the Jon Umstead Hospital for treatment (FE group). Another 20 subjects had presented for treatment of a psychotic relapse of schizophrenia (defined by DSM-IV) or schizoaffective disorder, related to their non-adherence to a prescribed outpatient treatment (RE group). No patient’s medication was discontinued as a condition for participation in the study. All available information from patients, family members, and treating clinicians indicated that each patient in the RE group had taken no antipsychotic medication for at least the preceding 2 weeks, and in most cases, much longer. All patients had at least two Positive symptom items rated > 4 at baseline on the Brief Psychiatric Rating Scale (BPRS) [17]. BPRS scores were also rated at the end of the study. Blood samples were collected before and after treatment with either ARI (n = 19) or RIS (n = 21).

#### Control group

We recruited race-matched, healthy control subjects (HC group) from the same communities as the subjects; they included hospital staff and their family members and friends (n = 29). Control subjects had no personal history of psychotic illness and no first or second degree relatives with psychotic illnesses.

### Antipsychotic treatment

All patients were randomized to receive one of two medications, RIS or ARI. The drugs were given to approximately equal subsets within both groups (FE and RE). The dose of antipsychotic medication for each individual was based on the judgment of a physician. RIS doses ranged from 1 to 6 mg/day, and ARI doses ranged from 5 to 30 mg/day. Subjects received treatment for approximately 2 weeks.

### Lipid sampling

All blood samples were collected in the early morning after overnight fasting. Baseline samples were drawn prior to starting antipsychotic treatment for the study. Subjects that had received any antipsychotic treatment during the referral process (e.g., due to agitation in an emergency department) were maintained untreated for 2–3 days before the baseline plasma sample was drawn. Follow-up samples were drawn after approximately 2 weeks of treatment, before discharge from the clinic.

Plasma lipid profiles were assessed (Lipomics Technologies, West Sacramento, CA) as described previously [18]. Briefly, plasma lipids were extracted, lipid classes isolated, and individual fatty acids within each lipid class quantified [19]. The analyses were based on concentrations (nmol/g plasma) of n3 and n6 free fatty acids and the numbers of individual n3 and n6 fatty acids contained in the phospholipids, PC and PE. Metabolites were designated names that reflected the lipid class and the fatty acid structure, e.g., palmitic acid in phosphatidylcholine was designated PC16: 0.

### Statistical analyses

For all variables, a correlation test for the hypothesis of normality was rejected for one or more of the groups at p<0.05 [20]. No common transformations altered this conclusion; thus, the data were found *not* to be approximately normal. Hence, all group pair-wise differences in lipid concentrations and product/precursor ratios were tested with Wilcoxon rank sum tests, except when comparing variables with covariate dependence.

#### Covariates

The demographic data in Table 1 shows that the FE and RE groups were not well-matched for gender with the HC group. Also, the FE group was not well-matched for age with the RE and HC groups. The group-effects can be confounded by these demographic covariates when both an imbalance is present across groups and the lipid variable is dependent on the covariate. Therefore, the possibility of confounding was addressed by checking Kendall’s tau rank correlations between each lipid and the covariates. Relevant covariates and interactions were first identified for each lipid variable using all three subject groups combined. The Pr(tau) was determined, under the null hypothesis of zero correlation between a given lipid variable and any covariate. Covariates included Age, Sex, Race, body mass index (BMI), Sex×Age (where × indicates an interaction between variables), Race×Sex, Race×Age, Race×Sex×Age, BMI×Sex, BMI×Race, and BMI×Sex×Race. Age and BMI were collinear; therefore, the covariate Age×BMI was not included. When the Pr(tau) < 0.05, the covariate effect on the lipid variable was separated from the group effect on this variable with a nonparametric analysis of covariance (nANCOVA), based on a rank-transformation procedure [21,22]. Thus, low nANCOVA p-values represented significant differences after adjusting for covariate dependencies. This rank-based method can capture monotonic nonlinear as well as linear covariate dependence. Furthermore, when all covariate interactions are included, it provides a conservative result when the lipid dependence upon a group is indistinguishable from the dependence upon a covariate due to covariate imbalances between groups. Therefore, we adjusted for all relevant covariates, and the differences between subject groups represent effects primarily due to disease or treatment.

**Table 1:** Demographics of subjects without (controls) or with schizophrenia (FE and RE), measured before (initial) and after (final) drug treatment.

	Controls (n=29)	FE + RE (n=40)	FE (n=20)	RE (n=20)
% Female	79.3^*^	27.5	35	20
% African American	82.7	72.5	65	80
Age (y)	41.0±9.5	31.8±12.2	27.0±9.8^*^	36.7±12.7
Height (in)	66.6±3.3	67.7±4.1	66.8±3.8	68.6±4.3
Weight (lbs) initial	186.1±48.7^a^	156.2±32.8	149.1±31.8	163.3±33.0
BMI (kg/m^2^) initial	29.4±7.0^a^	24±. 05.0	23.4±4.3	24.6±5.7
Waist circumference (in) initial	38.7±7.1	35.0±4.7	34.2±5.0	35.9±4.4
Weight (lbs) final		159.9±33	153.1±31.9	166.6±33.5
BMI (kg/m^2^) final		24.6±5.1	24.1±4.4	25.1±5.7
Waist circumference (in) final		36.1±5.1	35.1±5.4	37.1±4.7
BPRS initial		36.6±5.4	34.8±5.3^b^	38.4±5
BPRS final		25.6±6.4	22.8±5.9	28.4±5.8
Days treated		16.9±5.6	16.45±5.4	17.4±5.8

Values represent the mean ± standard deviation. FE, RE: patients with first episode or recurrent schizophrenia, respectively. BMI: body mass index. BPRS: Brief Psychiatric Rating Scale. *p<0.05, significantly different from the other two groups. ^a^p<0.05, Controls compared to FE; ^b^p<0.05, FE compared to RE

#### Lipid pathway analysis

Fatty acid product/precursor ratios were analyzed within each fatty acid synthesis pathway (n3 or n6), when they corresponded to a pure elongase or desaturase enzymatic reaction step [23]. However, precursors were not considered when over 10% of zeros (i.e., infinite ratios) were present within any subject group; otherwise, ratios with a value of 0 for the precursor were given the highest rank in our rank-based analysis.

#### Lipid concentration comparisons among groups

Corrections for multiple tests were set by assigning α=0.05 to each fatty acid pathway (i.e., n3 and n6) within each lipid class (i.e., PC, PE) and group comparison (HC vs. FE, HC vs. RE, FE vs. RE). The Bonferroni correction was applied for the number of fatty acid variables (B) within the particular pathway of interest. Differences between subject and control groups were considered significant at p<0.05/B, and they were considered trends at p<0.10/B, based on the Wilcoxon Rank Sum Test or McSweeney-Porter nANCOVA. Differences between pre- and post treatment values within a group were considered significant at p<0.05/B, and trends at p<0.10/B, based on the Wilcoxon Signed Ranks Test. Exploratory analyses, with larger numbers of tests, used both the conservative Bonferroni correction of alpha and control of the False Discovery Rate (FDR) [24,25]. Our lipid measures were not independent, but overwhelmingly exhibited positive monotonic mutual dependencies.

## Results

For the first set of analyses, samples from all 40 subjects with schizophrenia (FE + RE subjects) were compared with 29 race-matched HC subjects (Table 1). There were no significant differences in height, weight, BMI, or waist circumference between FE and RE groups. The HC group had significantly greater weight and BMI than the FE group. Age was not significantly different between the RE and HC groups. However, as expected, the FE group was significantly younger than both the HC (t-test, p=0.0014)) and RE (t-test, p= 0.01) groups. Also, as expected, the FE group had a lower mean total BPRS score (t-test, p=0.03) at enrollment than the RE group. The two subject groups (FE and RE) had similar gender compositions, but they were different from the gender composition of the HC group.

No demographic differences were found between the ARI and RIS treatment groups (not shown).

Linear regression analyses between the changes in the BPRS scores with drug treatment and changes in BMI, body weight, and waist circumferences showed correlation coefficients of 0.18409, -0.21112, and -0.03309, respectively. This indicated that the effect of drugs on disease severity (BPRS score) was not significantly correlated with the drug effects on subject characteristics.

### Metabolic Signature of Disease

The overall lipid classes at baseline were compared between subjects and controls (Table 2) and between subject groups. At baseline, the plasma concentrations of major lipid classes were not significantly different between control subjects and subjects with schizophrenia (FE + RE). There were no significant differences between HC and FE, HC and RE, or FE and RE. This was not surprising, because concentrations of the major lipid classes do not necessarily reflect differences in lipid composition, which depend on the individual lipid synthesis pathways.

**Table 2:** Baseline lipid concentrations in seven lipid classes for subjects without (controls) or with schizophrenia (FE and RE).

**Lipid Class**	**HC nmol/g median (1^st^ and 3^rd^ quartiles)**	**FE nmol/g median (1^st^ and 3^rd^ quartiles)**	**RE nmol/g median (1^st^ and 3^rd^ quartiles)**	**HC vs. FE p-value**	**HC vs. RE p-value**	**FE vs. RE p-value**
CE	2862 (2466, 3219)	2300 (2087, 2720)	2343 (2104, 2835)	0.676	0.597	0.524
LYPC	198.7 (160.5, 232.7)	205.6 (188.2, 227.6)	189.9 (165.0, 231.3)	0.474	0.607	0.314
PC	1472 (1383, 1592)	1400 (1275, 1665)	1349 (1269, 1742)	0.770	0.636	0.947
PE	140.9 (131.8, 174.7)	153.6 (131.8, 163.4)	142.0 (117.5, 170.2)	0.485	0.581	0.552
TG	677.1 (504.3, 906.2)	611.3 (472.3, 736.2)	771.4 (567.0, 1031)	0.380	0.437	0.157
DAG	34.31 (27.75, 56.41)	31.71 (23.92, 38.07)	39.66 (35.01, 47.12)	0.231	0.474	0.044
FA	321.3 (244.0, 575.7)	379.6 (231.4, 527.5)	421.2 (269.9, 489.4)	0.780	1.000	1.000

Concentrations represent median values (1^st^ and 3^rd^ quartiles) expressed in nmol/g of plasma. HC: healthy controls; FE, RE: patients with first episode or recurrent schizophrenia, respectively. Individual p-values for pair-wise comparisons were based on the Wilcoxon rank sum test, or the McSweeney-Porter nonparametric ANCOVA procedure (for analytes with covariate dependence), adjusted with the Bonferroni correction of alpha: 0.05/21 = 0.0024. CE: Cholesteryl esters; LYPC: lysophosphatidylcholine; PC: phosphatidylcholine; PE: phosphatidylethanolamine; TG: triacylglycerols; DAG: diacylglycerols; FA: fatty acids.

When we analyzed individual n3 and n6 lipids, we found significant differences between study groups in the compositions of PC and PE lipids (Table 3), both at baseline and after treatment. At baseline, the FE and HC groups showed significant differences in the PCn3 and PEn3 concentrations. In contrast, the FE and RE groups only showed significant differences in the PEn3 concentrations. After treatment, the PCn6, PEn3, and PEn6 concentrations were altered in the FE group, but not in the RE group.

**Table 3:** Lipid concentrations in Controls and Subjects with schizophrenia (FE and RE) at baseline (pretreatment) and after antipsychotic treatment (post treatment).

Lipid	Controls nmol/g	P values Baseline comparisons, FE v. HC, FE v.RE	First Episode			Recurrent Episode		
			Pre treatment nmol/g	Post treatment nmol/g	p-value, Pre v. Post	Pre treatment nmol/g	Post treatment nmol/g	p-value, Pre v. Post
PC.n3	155.6	0.0052, 0.0238	94.75	114.35	0.0484	122.6	121.7	0.1650
PC.n6	1293.5	0.1848, 0.6096	1051.05	1321.65	0.0056	1095.9	1206.1	0.1893
PE.n3	25	0.001^a^, 0.0032	15.45	21.35	0.0010*	23.4	20.9	0.2774
PE.n6	116	0.0696, 0.4217	86.25	115.85	0.0008*	99.1	103.05	0.7841

Concentrations represent median values (nmol/g of plasma); Controls or HC: healthy controls; First episode: drug-naïve patients with a first episode of schizophrenia; Recurrent episode: patients that had discontinued medication, and presented with recurrent schizophrenia. ^a^ Trend difference between first episode and control groups, McSweeney-Porter procedure, Bonferroni-corrected alpha for trends: p<0.10/63=0.0016; * significant difference, Wilcoxon signed ranks test, alpha adjusted with Bonferroni correction for significance: p<0.05/21=0.0024. Lipid notation indicates the lipid class and the double bond position, e.g., PC.n3 includes n3 fatty acids associated with PC phospholipids.

**Figure 1:**
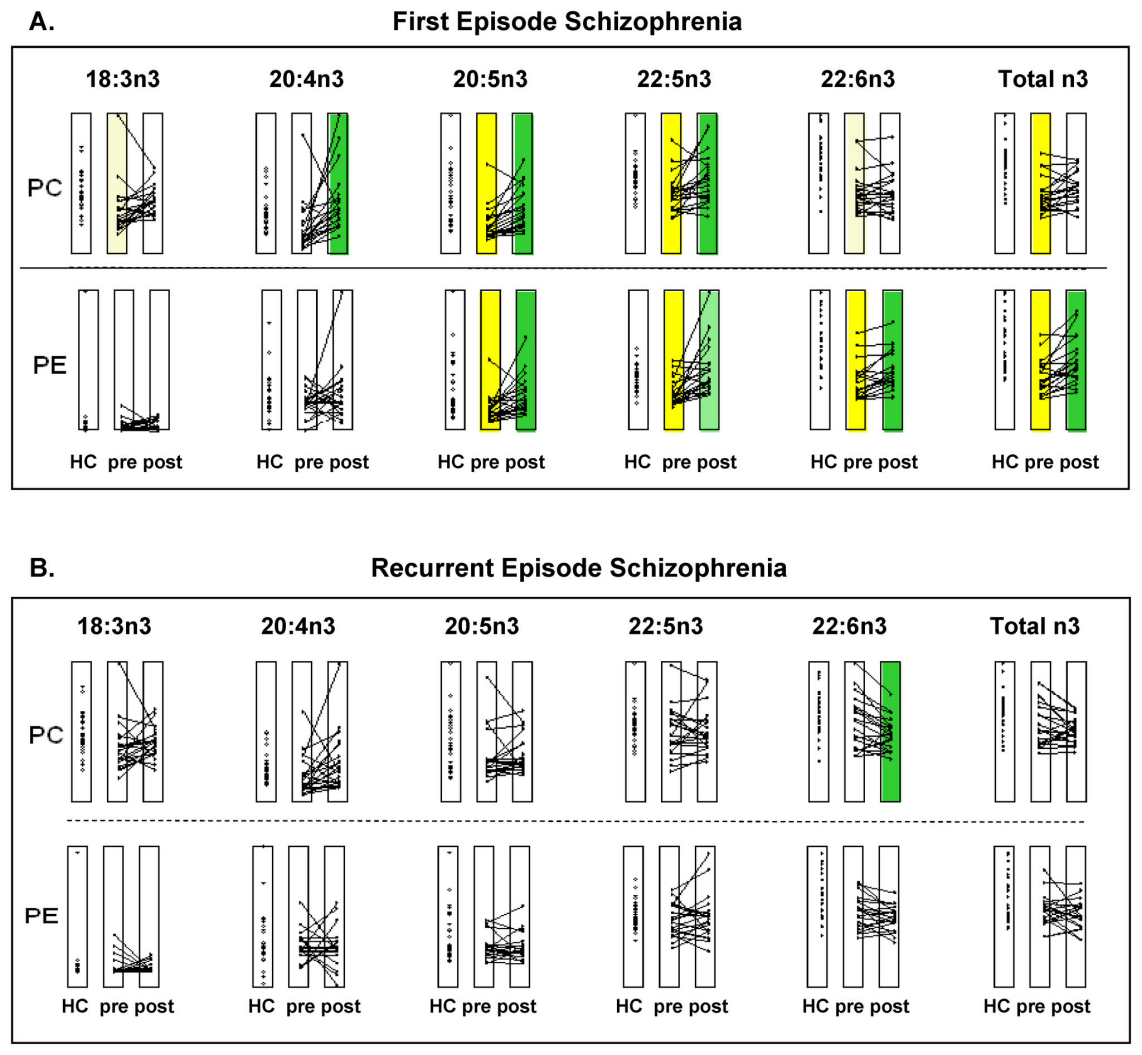
Alterations in plasma PC and PE n3 fatty acids in subjects with schizophrenia. Individual lipid profiles were compared between healthy controls (HC) and patients with (**A**) first episode (FE) or (**B**) recurrent (RE) schizophrenia (*Yellow*). Significant differences between HC and subjects with schizophrenia at baseline (pre-treatment) (*Green*). Significant differences within a group, before and after treatment. *pre*: pre-treatment values; *post*: post-treatment values. Lines indicate changes in individual subjects due to treatment. Data are scaled as a fraction of the maximum value for that lipid over all groups (HC, FE, and RE). *Pale color* intensity indicates a trend with corrected alpha values: p<0.10/7=0.014 (PE), p<0.10/6=0.017 (PC); *strong color* intensity indicates significance with corrected alpha values: p<0.05/7=0.0071 (PE), p<0.05/6=0.0083 (PC). Significant differences were evaluated as described in the Methods.

We next evaluated the individual n3 fatty acids (Figure 1 Table S1) incorporated in the individual PC and PE lipids. We found that some metabolites were significantly different between the HC group and the FE group before treatment (Figure 1, yellow shading). This indicated alterations in lipid metabolism that occurred early in schizophrenia. Drug treatment resulted in significant changes (pre- vs. postdifferences shown in green) in individual n3 lipids in the FE group (PC 20:4n3, 20:5n3, 22:5n3; PE 20:5n3, 22:5n3, 22:6n3). In contrast, the HC group and the RE group at baseline were not significantly different in n3 phospholipids (Figure 1 Table S1). Furthermore, unlike the FE group, the RE group showed little change in n3 phospholipids after drug treatment. Only one n3 (PC22: 6n3) showed a significant change with treatment in the RE group.

**Figure 2:**
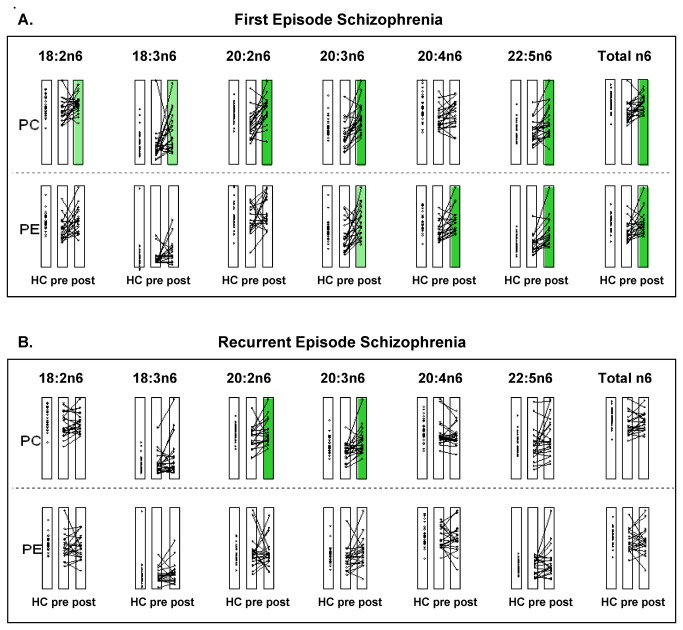
Alterations in plasma PC and PE n6 fatty acids in subjects with schizophrenia. Individual lipid profiles were compared between healthy controls (HC) and patients with (**A**) first episode (FE) or (**B**) recurrent (RE) schizophrenia (*Yellow*). Significant differences between HC and subjects with schizophrenia at baseline (pre-treatment) (*Green*). Significant differences within a group, before and after treatment. Lines indicate changes in individual subjects due to treatment. *pre*: pre-treatment values; *post*: post-treatment values. Data are scaled as a fraction of the maximum value for that lipid over all groups (HC, FE, and RE). *Pale color* intensity indicates a trend with corrected alpha values: p<0.10/8=0.0125 (PE and PC); *strong color* intensity indicates significance with corrected alpha values: p<0.05/8=0.00625 (PE and PC). Significant differences were evaluated as described in the Methods.

We also evaluated the n6 fatty acids (Figure 2) incorporated in the individual PC and PE lipids. We found that, at baseline, no significant differences were detected between the FE and the HC groups in the individual n6 fatty acids incorporated in PCs and PEs (Figure 2, top). However, in the FE group, drug treatment caused significant changes (pre vs. post) in the individual n6 lipids (PC 18:2n6, 18:3n6, 20:2n6, 20:3n6, 22:5n6; PE 20:3n6, 20:4n6, 22:5n6). Similarly, at baseline, the n6 phospholipids were not significantly different between the RE and the HC groups (Figure 2, bottom). In the RE group, drug treatment also caused significant changes, but in only two n6 lipids (20:2n6 and 20:3n6).

**Figure 3:**
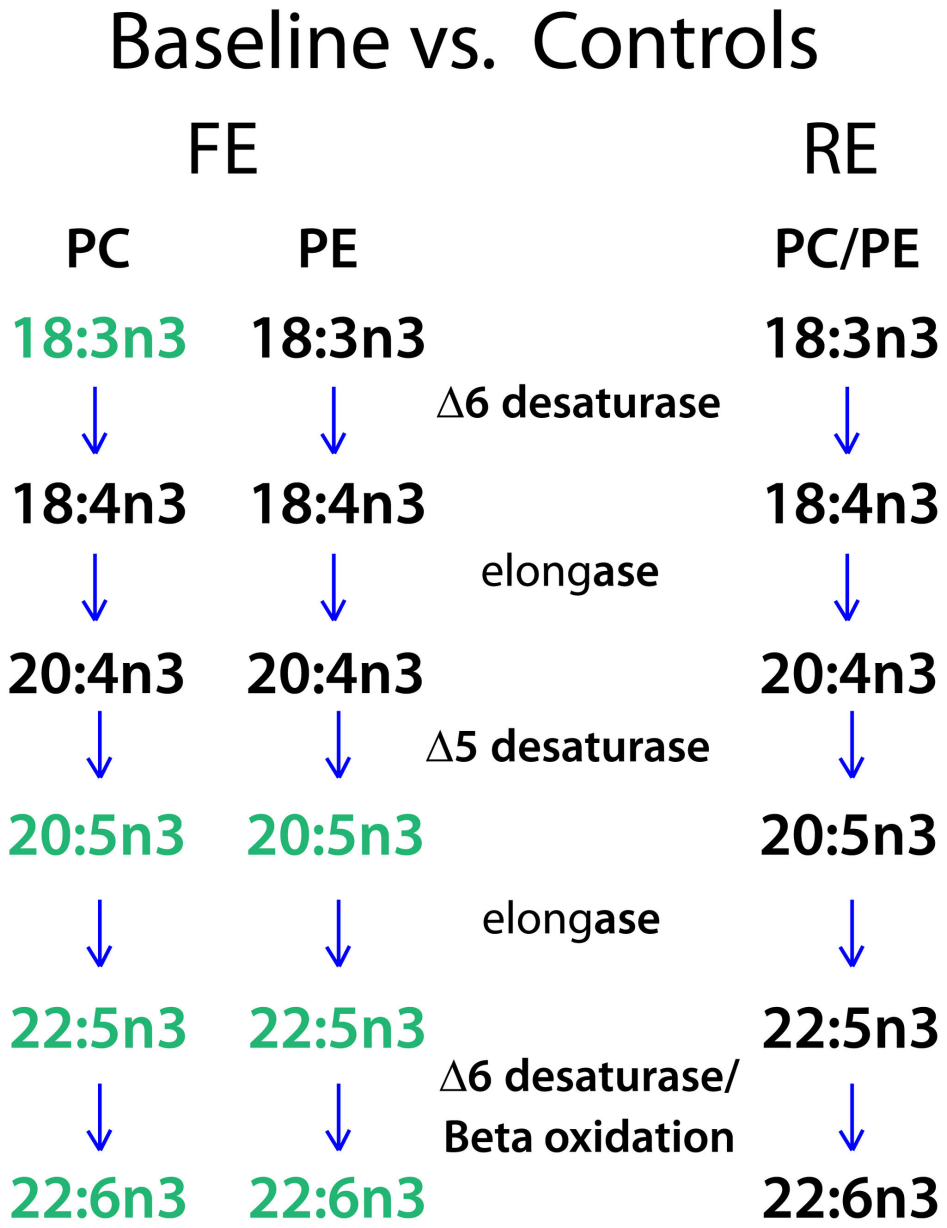
Changes in n3 fatty acid synthesis pathways with disease progression. Baseline levels of n3 fatty acid concentrations were compared between healthy controls (HC) and either subjects with first episode (FE, left) or recurrent (RE, right) schizophrenia. *Green* lettering indicates n3 fatty acids that were significantly increased compared to controls. Significant differences were evaluated as described in Methods.

To determine the implications of these findings, we investigated whether the changes in n3 and n6 fatty acids reflected changes in synthesis or degradation. We evaluated various steps of the n3 and n6 fatty acid synthesis pathways by examining the ratio of individual fatty acids that were considered the product and precursor of each enzyme in the synthesis pathway (Table 4). At baseline, only the FE and HC groups showed differences in fatty acid concentrations. Figure 3 illustrates how these differences affected the different steps in the n3 synthesis pathway. The highlighted fatty acids represent ratios that were different in FE compared to HC groups. For example, the ratio of PC22: 5n3 to PC20: 5n3 was significantly different between FE at baseline (median ratio =2.40) and HC (median ratio =1.53) (Wilcoxon Rank Sum Test, with corrected alpha: p<0.05/[17*3]=0.00098). In contrast, no differences in the fatty acid concentrations or ratios were detected between RE and HC groups.

**Table 4:** Comparison of baseline PC and PE synthesis pathways, based on product/precursor ratios of n3 and n6 lipids that represent enzymatic steps in controls (HC) and patients with schizophrenia (FE and RE).

	***Pretreatment***			***Posttreatment***	
***Product/precursor***	***HCratio***	***FEratio***	***REratio***	***FEratio***	***REratio***
***PC***					
22:6n3/22:5n3	3.977	3.418	3.707	2.474^#^	3.211^#^
20:5n3/20:4n3	7.167	7.292	7.867	4.944^#^	6.123
22.5n3/20.5n3	1.535	2.399*	1.804	1.712^#^	1.652
22:5n6/22:4n6	0.810	0.794	0.767	0.836	0.796
20:4n6/20:3n6	4.853	4.613	4.654	3.288^#^	3.710^#^
20:3n6/20:2n6	7.500	7.558	7.668	8.704	7.419
18:3n6/18:2n6	0.003	0.003	0.003	0.004	0.003
22:4n6/20:4n6	0.033	0.037	0.037	0.042	0.038
20:2n6/18:2n6	0.015	0.014	0.015	0.017^#^	0.017
***PE***					
22:6n3/22:5n3	3.882	3.226	4.054	2.594	3.262
22.5n3/20.5n3	3.133	3.829	3.050	3.00	3.342
22:5n6/22:4n6	0.636	0.639	0.652	0.850	0.743
20:4n6/20:3n6	15.137	15.269	15.500	12.375	12.105^#^
20:3n6/20:2n6	7.286	6.000	6.714	8.636	8.125
18:3n6/18:2n6	0.006	0.005	0.007	0.007	0.009
22:4n6/20:4n6	0.034	0.039	0.040	0.04	0.039
20:2n6/18:2n6	0.024	0.026	0.025	0.027	0.024

Values represent the medians of the concentration ratios measured at baseline in the groups indicated. HC: healthy controls; FE, RE: patients with first episode or recurrent schizophrenia, respectively. Group comparisons were evaluated with the McSweeney p-value to account for covariate dependence, or with the Wilcoxon Rank Sum test for lipids without covariate dependence. *p=0.00028, significantly different from HC, based on Wilcoxon Rank Sum test p-value, after adjusting with Bonferroni alpha: p<0.05/(17*3)=0.00098. ^#^p<0.00058, significantly different from pretreatment, based on Wilcoxon Rank Sum test p-value, after adjusting with Bonferroni alpha: p<0.05/(17*2)=0.00147.

**Figure 4:**
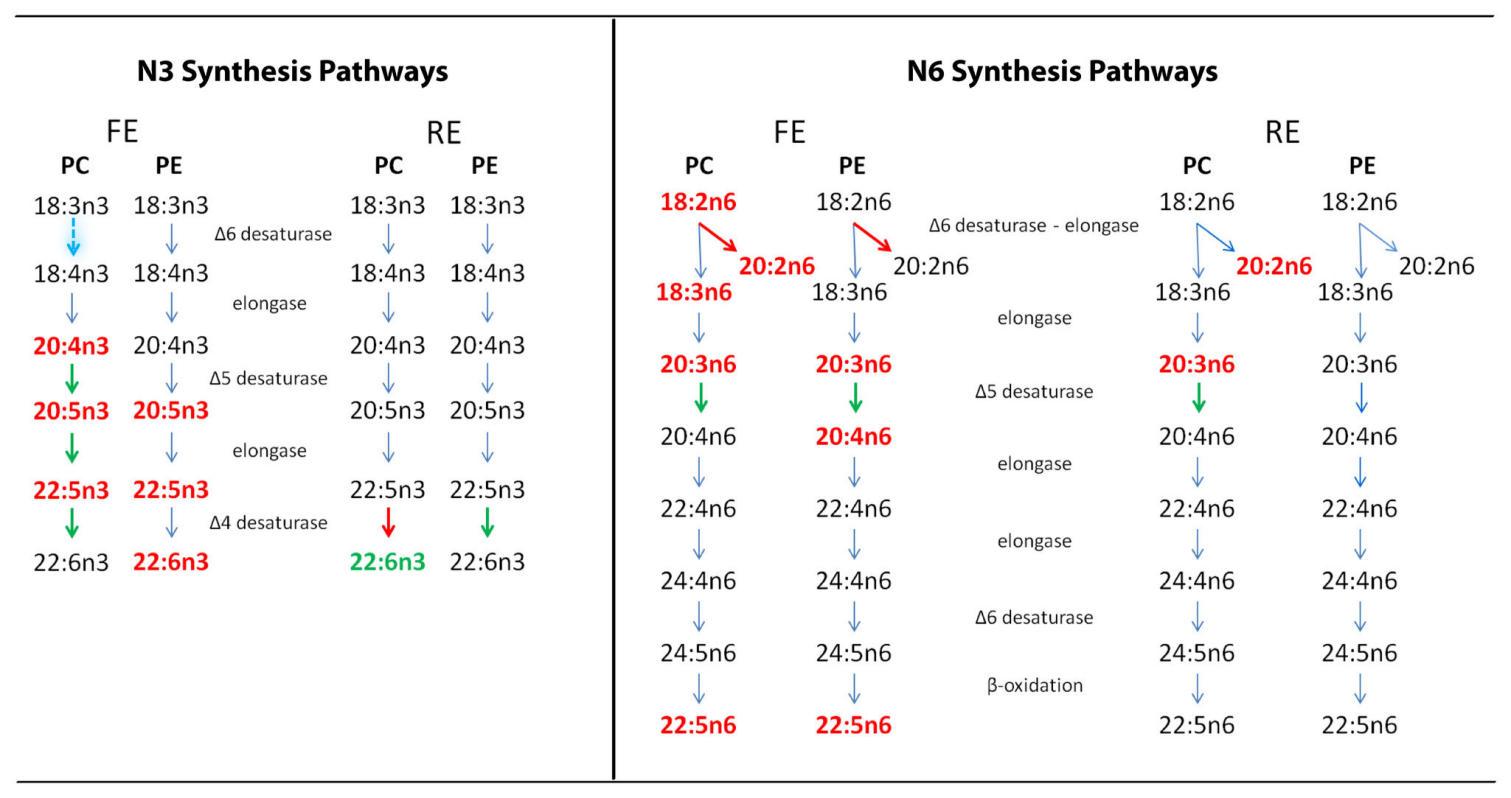
Antipsychotic treatment effects on n3 and n6 fatty acid synthesis pathways. Fatty acid concentrations were compared before and after treatment in subjects with first episode (FE) or recurrent (RE) schizophrenia. *Green* and *red* lettering represent fatty acids that showed significant increases and reductions, respectively, with treatment. Green and red arrows represent enzymatic activities that showed significant increases and reductions, respectively, with treatment. Significant differences were evaluated as described in Methods.

We then evaluated the drug-induced changes in the ratios of fatty acid products to precursors in both the n3 and n6 fatty acid synthesis pathways (Table 4). This analysis would determine which enzymes were modulated by drug treatment. We compared changes in lipids for patients treated with ARI to those treated with RIS, and found no significant differences. Therefore, we pooled the treatment data for further analyses. Figure 4 illustrates how the drug-induced changes in n3 and n6 fatty acids affected the n3 and n6 synthesis pathways. The highlighted fatty acids indicate those that were significantly different before and after treatment. The fatty acid ratio analyses showed that the antipsychotic treatments significantly affected both the n3 and n6 fatty acid synthesis pathways for both FE and RE groups.

Schizophrenia is associated with a high prevalence of metabolic syndrome and with a state of chronic, low-grade inflammation; moreover, these conditions might be interrelated [26]. The n3 and n6 fatty acid synthesis pathways are important for maintaining the balance between anti- and pro-inflammatory conditions. Therefore, differences in the n3/n6 ratio contained in lipids may influence the levels of low-grade inflammation (or be a symptom of disease) in the different study groups. We found that patients with FE had lower n3/n6 ratios than either RE or HC groups. Furthermore, when patients with FE received antipsychotic treatment, the n3/n6 ratios were restored to HC levels. In contrast, the n3/n6 ratios were similar in the RE and HC groups, and the ratios did not improve with treatment.

Our covariate analysis ensured that the effects we observed were independent of differences in body weight and age. Thus, it seems likely that the changes in fatty acid synthesis that we observed were relevant to the stage of the disease.

### Exploratory study of seven lipid classes

In addition to our primary focus, which was to study the n3 and n6 fatty acids within the major phospholipid classes (PC and PE), we conducted an exploratory study to determine whether the different study groups (at baseline) showed differences in the fatty acid profiles for seven lipid classes, i.e., PC, PE, cholesteryl ester (CE), lysoPC (LYPC), triacylglycerol (TAG), diacyglycerol, and free fatty acids (FFA). We compared baseline profiles between FE and HC and between RE and HC, but significant results were found only for the FE vs. HC comparison. We used the 10% FDR control, which is generally appropriate for exploratory work. In the FE group, 10 lipids were present at lower concentrations in than in the HC group; five involved 20:5n3 (in CE, PC, PE, TAG, and FFA classes); two involved 22:5n3 (in PC and PE classes); and two involved 22:6n3 (in LYPC and PE classes). The largest significant difference was in the total n3 content of PE, which was much lower in FE than in HC. We also controlled for a Type I error with the Bonferroni correction applied to alpha values of 0.10 and 0.05. A few of these tests were significant at the p<0.05 level (corrected). Thus, the difference between FE and HC groups at baseline was evident across several lipid classes.

## Discussion

In this study, the subjects with schizophrenia (FE and RE) did not show significant differences from control subjects in overall levels of lipids in the seven classes (i.e., PC, PE, CE, LYPC, etc). However, differences could be detected when individual n3 and n6 phospholipids were compared between groups. One major finding was that the n3 phospholipid concentrations in FE subjects were significantly different from those in HC subjects; however, the n6 phospholipid concentrations were similar between FE and HC groups. These findings were taken to reflect the effects of the disease, because the results were similar, whether we used a broader analysis or whether we subtracted the effects of many relevant confounders that might influence the differences, particularly body weight, age, and gender (Table S1). Thus, our findings suggested that alterations in n3 lipid metabolism occurred early in the disease. In contrast, the RE subjects exhibited no significant differences in n3 or n6 phospholipids compared to HC subjects. This suggested that either disease progression or previous drug treatment ameliorated the early alterations in lipid metabolic pathways associated with schizophrenia.

Another major finding was that antipsychotic drug treatment in the FE group caused significant changes (pre- vs. post-treatment) in both the n3 and n6 lipid concentrations. However, in the RE group, drug treatment caused few changes in phospholipids. Again, these results reflected significant differences after adjusting for body weight, age, and gender (although age was not significantly different between RE and HC). Thus, the results suggested that the effects of RIS and ARI on lipid metabolism were altered, either by disease progression or by previous treatment with drugs.

The finding that changes in lipid metabolism occurred early in the course of schizophrenia was consistent with Horrobin’s original hypothesis [27] and with earlier studies in red blood cells [28] and two recent meta-analyses [29,30]. This notion was also supported by in vivo brain studies, based on ^31^P magnetic resonance (MR) spectroscopy data, which also showed that membrane phospholipids were altered in neurons in the prefrontal cortex of adolescent relatives that were at genetic risk for schizophrenia [31]. The metabolomics findings of this study were also congruent with other disparate strands of evidence that have implicated a neurodevelopmental basis to schizophrenia [32]. Interestingly, early disease-related changes were previously postulated to reflect an accelerated breakdown in membrane phospholipids that may underlie developmentally-mediated synaptic pruning processes that may play a role in schizophrenia [33].

We used ratios of the fatty acids that were considered the product and precursor of each enzyme in the synthesis pathway to evaluate potential changes in the individual steps of the fatty acid synthesis pathways. While this method will not provide an exact estimation of the enzymatic activity in an individual tissue, it does allow an estimate of the average change in enzymes from all tissues that contribute to the plasma fatty acid pool. The results from the analysis of fatty acid ratios suggested that FE subjects had altered elongase activity compared to controls. The different profiles of the FE vs. HC groups suggested that early schizophrenia was associated with altered levels of EPA (20:5n3) and docosahexaenoic acid (DHA; 22:6n3). This indicated that the Δ5 desaturase activity may be altered in the FE group compared to controls. Because the same effect was observed in both PC and PE pathways, it is likely that the disease caused an increase in synthesis rather than a reduction in degradation. However, given that only the n3, and *not* the n6, lipid pathways were different between FE and HC groups, it seems clear that lipid degradation may also be a contributing factor.

At many steps during synthesis, individual n3 and n6 fatty acids must compete for enzymes that function in both pathways. If the fatty acid synthesis pathway is taken into consideration, in the FE group, drug treatment appeared to affect all the fatty acids in unison. Therefore, it is possible that all the enzymes in the pathways were upregulated, and this caused reduced concentrations of intermediate metabolites (precursors) in both pathways. In contrast, in the RE group, only a few enzymes appeared to be affected by drug treatment.

Our finding that the medication-naïve FE group had alterations in both the n3 and n6 pathways was consistent with previous studies on lipid metabolic alterations involved in schizophrenia [34]. For example, a previous study found that a deficiency in n3 fatty acids was associated with decreased dopamine concentrations and prefrontal D2 receptors [35]. Furthermore, both n3 and n6 fatty acids were found to differentially affect the binding of serotonin (5-HT) to receptors and transporters in rat brain [36]. It was also observed that n3 fatty acids, like DHA, could influence the turnover rate of 5-HT in rat brain [37], and this was associated with changes in cognitive behavior. Moreover, the cerebrospinal fluid levels of 5-hydroxyindoleacetic acid (5-HIAA, a metabolite of 5-HT) were correlated with plasma DHA levels in healthy volunteers [38].

The present results were consistent with our previous observations [11] that subjects with schizophrenia exhibited reduced n3 PUFAs in PC and PE compared to controls. Our previous study included subjects with both FE and RE schizophrenia, but we had not separated the two groups; therefore, those results may have been more reflective of the FE group. The present study also demonstrated that the results found in the FE group were independent of age and body weight; therefore, the present results were less likely to be biased by subject selection.

Prior studies have shown altered lipid metabolism in schizophrenia, particularly in the PCs and PEs present in erythrocytes [39], post-mortem brains [40], and living brain (*in vivo* MR spectroscopy studies) [41]. Other studies showed that, compared to HC subjects, phospholipid n3 fatty acids were reduced in either RE or FE subjects [42]. The present study demonstrated that, in the absence of medication, the n3 fatty acids in PEs were significantly different between FE and RE groups. However, it remains unclear whether the mechanisms that lead to reduced n3 PUFAs early in the course of illness are the same as those that operate in the later stages.

We also found that subjects with FE schizophrenia exhibited more sensitivity to drug treatment than those with RE. The FE group showed significant drug-induced changes in six n3 (PC 20:4n3, 20:5n3, 22:5n3; PE 20:5n3, 22:5n3, 22:6n3; Figure 1) and eight n6 (PC 18:2n6, 18:3n6, 20:2n6, 20:3n6, 22:5n6; PE 20:3n6, 20:4n6, 22:5n6; Figure 2) phospholipids; the RE group showed significant changes in only one n3 (PC22: 6n3; Figure 1) and two n6 (PC 20:2n6, 20:3n6; Figure 2) phospholipids. In both groups, the drugs appeared to have somewhat stronger effects on the n6 than on the n3 content of phospholipids. Previous studies showed that treatments with other atypical antipsychotics, olanzapine and clozapine, were associated with increases in 20:4n6 in subjects with schizophrenia [43,44]. A recent study by Sumiyoshi et al. [45] also demonstrated that psychological assessments with the SAPS and SANS test scores showed improvements in subjects with chronic schizophrenia after treatment with atypical antipsychotic drugs (either olanzapine or perospirone). Taken together, the results suggested that antipsychotic drugs may play a key role in lipid homeostasis in schizophrenia by regulating fatty acid biosynthesis.

As part of the analysis in the present study, we accounted for differences in age, gender, body weight, etc. among the HC, FE, and RE groups. However, it is possible that other environmental factors, like diet, might play an important role in the PUFA deficits observed in schizophrenia. One study indicated that subjects with schizophrenia tended to consume the same or higher levels of n3 and n6 PUFAs compared to healthy controls [46]. It is not known whether the dietary findings in subjects with chronic schizophrenia and long treatment histories have the same implications for subjects with FE schizophrenia. In the present study, we assumed that any persistent dietary differences might be reflected by differences in BMI and body weight between groups, which were accounted for in the covariate adjustments. Thus, we presumed that the observations in the present study were unlikely to be due to dietary differences alone, although this possibility cannot be ruled out. Future investigations should undertake a comprehensive analysis of dietary intake.

Finally, the observations in our study may be relevant to novel treatment approaches. Recent studies have shown that n3 supplementation may be neuroprotective. For example, when subjects with schizophrenia received supplementation with EPA (n3), the 5-HT-mediated amplification of platelet aggregation was markedly enhanced [47]; this suggested that membrane PUFA deficits might be associated with the blunted serotonergic platelet responsivity observed in schizophrenia. Moreover, when n3 supplementation was combined with antipsychotic therapy, subtle MR imaging changes were observed in subjects with FE schizophrenia [48]; this suggested that n3 fatty acid intake may offer a therapeutic benefit early in the course of psychosis [49]. However, our findings suggested that n3 supplementation may not be effective in subjects with RE schizophrenia.

Our results showed that drug treatment in the FE group appeared to have effects on all n3 and n6 fatty acids. This suggested that all the enzymes in the synthesis pathways were up-regulated. However, because individual n3 and n6 fatty acids are synthesized in competitive processes by enzymes that function in both pathways, the drug may have increased enzyme activity in one pathway at the expense of producing intermediates in the other pathway. The implications of these treatment-induced changes remain to be determined.

As discussed earlier, schizophrenia was associated with increased inflammation [26]; the n3 and n6 fatty acids play important roles in the balance of anti- and pro-inflammatory processes. For example, arachidonic acid (20:4n6), which leads to rapid production of inflammatory cytokines, competes for the same enzymes with EPA (20:5n3), which slows the production of inflammatory cytokines. DHA (22:6n3) also reduces inflammatory potential. It is therefore possible that, at least in part, the benefits and/or side effects of treatment may be related to changes in the n3 and n6 fatty acids, and these effects may vary for different medications. Future studies with larger samples and over longer periods of time are needed to address this important question.

## Supporting Information

Table S1 Lipid concentrations were compared to determine significant differences between controls (HC) and patients with schizophrenia (FE and RE). Concentrations are shown for lipids in the major lipid classes studied and for individual fatty acids (species) within each lipid class.
(DOC)

## References

[1] Rice DP (1999) The economic impact of schizophrenia. J Clin Psychiatry 60 Suppl 1: 4-6; discussion 28-30. PubMed : 10037163 10037163

[2] McEvoy JP (2007) The costs of schizophrenia. J Clin Psychiatry 68 Suppl 14: 4-7. doi:10.4088/JCP.0207e04. PubMed: 18284271. 18284271

[3] Kane JM, Potkin SG, Daniel DG, Buckley PF (2011) A double-blind, randomized study comparing the efficacy and safety of sertindole and risperidone in patients with treatment-resistant schizophrenia. J Clin Psychiatry 72: 194-204. doi:10.4088/JCP.07m03733yel. PubMed: 20673553. 20673553 10.4088/JCP.07m03733yel

[4] Cohen D, Dekker JJ, Peen J, Gispen-de Wied CC (2006) Prevalence of diabetes mellitus in chronic schizophrenic inpatients in relation to long-term antipsychotic treatment. Eur Neuropsychopharmacol 16: 187-194. doi:10.1016/S0924-977X(06)70080-3. PubMed: 16263247. 16263247 10.1016/j.euroneuro.2005.09.003

[5] Mukherjee S, Decina P, Bocola V, Saraceni F, Scapicchio PL (1996) Diabetes mellitus in schizophrenic patients. Compr Psychiatry 37: 68-73. doi:10.1016/S0010-440X(96)90054-1. PubMed: 8770530. 8770530 10.1016/s0010-440x(96)90054-1

[6] American Diabetes Association, American Psychiatric Association, American Association of Clinical Endocrinologists, North American Association for the Study of Obesity (2004) Consensus development conference on antipsychotic drugs and obesity and diabetes. J Clin Psychiatry 65: 267-272. doi:10.4088/JCP.v65n0219. PubMed: 15003083. 15003083 10.4088/jcp.v65n0219

[7] McQuade RD, Stock E, Marcus R, Jody D, Gharbia NA et al. (2004) A comparison of weight change during treatment with olanzapine or aripiprazole: results from a randomized, double-blind study. J Clin Psychiatry 65 Suppl 18: 47-56. PubMed: 15600384. 15600384

[8] Saddichha S, Manjunatha N, Ameen S, Akhtar S (2008) Diabetes and schizophrenia - effect of disease or drug? Results from a randomized, double-blind, controlled prospective study in first-episode schizophrenia. Acta Psychiatr Scand 117: 342-347. doi:10.1111/j.1600-0447.2008.01158.x. PubMed: 18307585. 18307585 10.1111/j.1600-0447.2008.01158.x

[9] Sengupta S, Parrilla-Escobar MA, Klink R, Fathalli F, Ying Kin N et al. (2008) Are metabolic indices different between drug-naive first-episode psychosis patients and healthy controls? Schizophr Res 102: 329-336. doi:10.1016/j.schres.2008.02.013. PubMed: 18396386. 18396386 10.1016/j.schres.2008.02.013

[10] Horrobin DF (1998) The membrane phospholipid hypothesis as a biochemical basis for the neurodevelopmental concept of schizophrenia. Schizophr Res 30: 193-208. doi:10.1016/S0920-9964(97)00151-5. PubMed: 9589514. 9589514 10.1016/s0920-9964(97)00151-5

[11] Kaddurah-Daouk R, McEvoy J, Baillie RA, Lee D, Yao JK et al. (2007) Metabolomic mapping of atypical antipsychotic effects in schizophrenia. Mol Psychiatry 12: 934-945. doi:10.1038/sj.mp.4002000. PubMed: 17440431. 17440431 10.1038/sj.mp.4002000

[12] Hannun YA, Obeid LM (2008) Principles of bioactive lipid signalling: lessons from sphingolipids. Nat Rev Mol Cell Biol 9: 139-150. doi:10.1038/nrm2329. PubMed: 18216770. 18216770 10.1038/nrm2329

[13] Luo C, Wang K, Liu, de Q, Li Y, Zhao QS (2008) The functional roles of lipid rafts in T cell activation, immune diseases and HIV infection and prevention. Cell Mol Immunol 5: 1-7. doi:10.1038/cmi.2008.1. PubMed: 18318989. 18318989 10.1038/cmi.2008.1 PMC4652918

[14] Zeman M, Jirak R, Vecka M, Raboch J, Zak A. (2012) N-3 polyunsaturated fatty acids in psychiatric diseases: Mechanisms and clinical data. Neuro Endocrinol Lett 33: 736–48. PubMed: 23391975. 23391975

[15] Farooqui AA, Horrocks LA, Farooqui T (2007) Modulation of inflammation in brain: a matter of fat. J Neurochem 101: 577-599. PubMed: 17257165. 17257165 10.1111/j.1471-4159.2006.04371.x

[16] Kaddurah-Daouk R, McEvoy J, Baillie R, Zhu H, Ky J et al. (2012) Impaired plasmalogens in patients with schizophrenia. Psychiatry Res, 198: 347–52. PubMed: 22513041. 22513041 10.1016/j.psychres.2012.02.019

[17] Rhoades HM, Overall JE (1988) The semistructured BPRS interview and rating guide. Psychopharmacol Bull 24: 101-104. PubMed: 3290934. 3290934

[18] Watkins SM, Reifsnyder PR, Pan HJ, German JB, Leiter EH (2002) Lipid metabolome-wide effects of the PPARgamma agonist rosiglitazone. J Lipid Res 43: 1809-1817. doi:10.1194/jlr.M200169-JLR200. PubMed: 12401879. 12401879 10.1194/jlr.m200169-jlr200

[19] Watkins SM, Lin TY, Davis RM, Ching JR, DePeters EJ et al. (2001) Unique phospholipid metabolism in mouse heart in response to dietary docosahexaenoic or alpha-linolenic acids. Lipids 36: 247-254. doi:10.1007/s11745-001-0714-8. PubMed: 11337979. 11337979 10.1007/s11745-001-0714-8

[20] Johnson RA, Wichern DW (1998) Applied Multivariate Statistical Analysis. 4th ed. Hall: Prentice p. 193, 222-223.

[21] Barrett TJ (2011) Computations using Analysis of Covariance. WIREs. Comput Stat 3: 260-268. doi:10.1002/wics.165.

[22] McSweeney M, Porter AC (1971) Small sample properties of nonparametric index of response and rank analysis of covariance. Occasional paper No 16 East Lansing, MI: Michigan State University, Office of Research Consultation.

[23] Gregory MK, Gibson RA, Cook-Johnson RJ, Cleland LG, James MJ (2011) Elongase Reactions as Control Points in Long-Chain Polyunsaturated Fatty Acid Synthesis. PLOS ONE 6: e29662. doi:10.1371/journal.pone.0029662. PubMed: 22216341. 22216341 10.1371/journal.pone.0029662 PMC3245304

[24] Benjamini Y, Hochberg Y (1995) Controlling the False Discovery Rate: A Practical and Powerful Approach to Multiple Testing. J R Stat Soc B Stat Methodol 57: 289-300.

[25] Benjamini Y, Yekutieli D (2001) The control of the false discovery rate in multiple testing under dependency. Ann Statist 29: 1165-1188. doi:10.1214/aos/1013699998.

[26] Leonard BE, Schwarz M, Myint AM (2012) The metabolic syndrome in schizophrenia: is inflammation a contributing cause? J Psychopharmacol 26: 33-41. doi:10.1177/0269881111431622. PubMed: 22472311. 22472311 10.1177/0269881111431622

[27] Horrobin DF, Huang YS (1983) Schizophrenia: the role of abnormal essential fatty acid and prostaglandin metabolism. Med Hypotheses 10: 329-336. doi:10.1016/0306-9877(83)90119-6. PubMed: 6348496. 6348496 10.1016/0306-9877(83)90119-6

[28] Peet M, Laugharne J, Rangarajan N, Horrobin D, Reynolds G (1995) Depleted red cell membrane essential fatty acids in drug-treated schizophrenic patients. J Psychiatr Res 29: 227-232. doi:10.1016/0022-3956(95)00001-L. PubMed: 7473298. 7473298 10.1016/0022-3956(95)00001-l

[29] Hoen WP, Lijmer JG, Duran M, Wanders RJ, van Beveren NJ et al. (2012) Red blood cell polyunsaturated fatty acids measured in red blood cells and schizophrenia: A meta-analysis. Psychiatry Res, 207: 1–12. PubMed: 23068078. 23068078 10.1016/j.psychres.2012.09.041

[30] van der Kemp WJ, Klomp DW, Kahn RS, Luijten PR, Hulshoff Pol HE (2012) A meta-analysis of the polyunsaturated fatty acid composition of erythrocyte membranes in schizophrenia. Schizophr Res 141: 153-161. doi:10.1016/j.schres.2012.08.014. PubMed: 22981812. 22981812 10.1016/j.schres.2012.08.014

[31] Keshavan MS, Stanley JA, Montrose DM, Minshew NJ, Pettegrew JW (2003) Prefrontal membrane phospholipid metabolism of child and adolescent offspring at risk for schizophrenia or schizoaffective disorder: an in vivo 31P MRS study. Mol Psychiatry 8: 316-323, 251. doi:10.1038/sj.mp.4001325. PubMed: 12660804. 12660804 10.1038/sj.mp.4001325

[32] Fatemi SH, Folsom TD (2009) The neurodevelopmental hypothesis of schizophrenia, revisited. Schizophr Bull 35: 528-548. doi:10.1093/schbul/sbn187. PubMed: 19223657. 19223657 10.1093/schbul/sbn187 PMC2669580

[33] Keshavan MS, Anderson S, Pettegrew JW (1994) Is schizophrenia due to excessive synaptic pruning in the prefrontal cortex? The Feinberg hypothesis revisited. J Psychiatr Res 28: 239-265. doi:10.1016/0022-3956(94)90009-4. PubMed: 7932285. 7932285 10.1016/0022-3956(94)90009-4

[34] Mahadik SP, Evans DR (2003) Is schizophrenia a metabolic brain disorder? Membrane phospholipid dysregulation and its therapeutic implications. Psychiatr Clin North Am 26: 85-102. doi:10.1016/S0193-953X(02)00033-3. PubMed: 12683261. 12683261 10.1016/s0193-953x(02)00033-3

[35] Ohara K (2007) The n-3 polyunsaturated fatty acid/dopamine hypothesis of schizophrenia. Prog Neuropsychopharmacol Biol Psychiatry 31: 469-474. doi:10.1016/j.pnpbp.2006.11.013. PubMed: 17184889. 17184889 10.1016/j.pnpbp.2006.11.013

[36] du Bois TM, Deng C, Bell W, Huang XF (2006) Fatty acids differentially affect serotonin receptor and transporter binding in the rat brain. Neuroscience 139: 1397-1403. doi:10.1016/j.neuroscience.2006.02.068. PubMed: 16600514. 16600514 10.1016/j.neuroscience.2006.02.068

[37] Dervola KS, Roberg BA, Wøien G, Bogen IL, Sandvik TH et al. (2012) Marine omega-3 polyunsaturated fatty acids induce sex-specific changes in reinforcer-controlled behaviour and neurotransmitter metabolism in a spontaneously hypertensive rat model of ADHD. Behav Brain Funct 8: 56. doi:10.1186/1744-9081-1188-1156. PubMed: 23228189. 23228189 10.1186/1744-9081-8-56 PMC3573936

[38] Hibbeln JR, Umhau JC, Linnoila M, George DT, Ragan PW et al. (1998) A replication study of violent and nonviolent subjects: cerebrospinal fluid metabolites of serotonin and dopamine are predicted by plasma essential fatty acids. Biol Psychiatry 44: 243-249. doi:10.1016/S0006-3223(98)00143-7. PubMed: 9715355. 9715355 10.1016/s0006-3223(98)00143-7

[39] Keshavan MS, Mallinger AG, Pettegrew JW, Dippold C (1993) Erythrocyte membrane phospholipids in psychotic patients. Psychiatry Res 49: 89-95. doi:10.1016/0165-1781(93)90032-C. PubMed: 8140184. 8140184 10.1016/0165-1781(93)90032-c

[40] Yao JK, Leonard S, Reddy RD (2000) Membrane phospholipid abnormalities in postmortem brains from schizophrenic patients. Schizophr Res 42: 7-17. doi:10.1016/S0920-9964(99)00095-X. PubMed: 10706981. 10706981 10.1016/s0920-9964(99)00095-x

[41] Potwarka JJ, Drost DJ, Williamson PC, Carr T, Canaran G et al. (1999) A 1H-decoupled 31P chemical shift imaging study of medicated schizophrenic patients and healthy controls. Biol Psychiatry 45: 687-693. doi:10.1016/S0006-3223(98)00136-X. PubMed: 10187998. 10187998 10.1016/s0006-3223(98)00136-x

[42] Arvindakshan M, Sitasawad S, Debsikdar V, Ghate M, Evans D et al. (2003) Essential polyunsaturated fatty acid and lipid peroxide levels in never-medicated and medicated schizophrenia patients. Biol Psychiatry 53: 56-64. doi:10.1016/S0006-3223(02)01443-9. PubMed: 12513945. 12513945 10.1016/s0006-3223(02)01443-9

[43] Glen AI, Cooper SJ, Rybakowski J, Vaddadi K, Brayshaw N et al. (1996) Membrane fatty acids, niacin flushing and clinical parameters. Prostaglandins Leukot Essent Fatty Acids 55: 9-15. doi:10.1016/S0952-3278(96)90139-8. PubMed: 8888117. 8888117 10.1016/s0952-3278(96)90139-8

[44] Horrobin DF (1999) The effects of antipsychotic drugs on membrane phospholipids: a possible novel mechanism of action of clozapine. In: Peet M Glen I Horrobin DF Phospholipid spectrum disorder in psychiatry. Carnforth: Marius Press pp. 113-117.

[45] Sumiyoshi T, Higuchi Y, Matsui M, Itoh H, Uehara T et al. (2011) Membrane fatty acid levels as a predictor of treatment response in chronic schizophrenia. Psychiatry Res 186: 23-27. doi:10.1016/j.psychres.2010.07.049. PubMed: 20800904. 20800904 10.1016/j.psychres.2010.07.049

[46] Strassnig M, Singh Brar J, Ganguli R (2005) Dietary fatty acid and antioxidant intake in community-dwelling patients suffering from schizophrenia. Schizophr Res 76: 343-351. doi:10.1016/j.schres.2005.03.002. PubMed: 15949667. 15949667 10.1016/j.schres.2005.03.002

[47] Yao JK, Magan S, Sonel AF, Gurklis JA, Sanders R et al. (2004) Effects of omega-3 fatty acid on platelet serotonin responsivity in patients with schizophrenia. Prostaglandins Leukot Essent Fatty Acids 71: 171-176. doi:10.1016/j.plefa.2004.03.011. PubMed: 15253886. 15253886 10.1016/j.plefa.2004.03.011

[48] Wood SJ, Cocchi L, Proffitt TM, McConchie M, Jackson GD et al. (2010) Neuroprotective effects of ethyl-eicosapentaenoic acid in first episode psychosis: a longitudinal T2 relaxometry pilot study. Psychiatry Res 182: 180-182. doi:10.1016/j.pscychresns.2009.12.003. PubMed: 20413278. 20413278 10.1016/j.pscychresns.2009.12.003

[49] Amminger GP, Schäfer MR, Papageorgiou K, Klier CM, Cotton SM et al. (2010) Long-chain omega-3 fatty acids for indicated prevention of psychotic disorders: a randomized, placebo-controlled trial. Arch Gen Psychiatry 67: 146-154. doi:10.1001/archgenpsychiatry.2009.192. PubMed: 20124114. 20124114 10.1001/archgenpsychiatry.2009.192

