# Supplementary material for: Lipidomics Reveals Early Metabolic Changes in Subjects with Schizophrenia: Effects of Atypical Antipsychotics
Source: PLoS One. 2013 Jul 24;8(7):e68717. doi: 10.1371/journal.pone.0068717 (PMC3722141; doi:10.1371/journal.pone.0068717)
Supplement: Table S1 — Concentrations are shown for lipids in the major lipid classes studied and for individual fatty acids (species) within each lipid class. (DOC) [file pone.0068717.s001.doc]

Supplementary Table S1. Lipid concentrations were compared to determine significant differences between controls (HC) and patients with schizophrenia (FE and RE). Concentrations are shown for lipids in the major lipid classes studied and for individual fatty acids (species) within each lipid class.

|  | |  | | | **HC v. FE** | | **HC v. RE** | |  |
| --- | --- | --- | --- | --- | --- | --- | --- | --- | --- |
| **Lipids** | | **HC** | **FE** | **RE** | **Wilc** | **McSw** | **Wilc** | **McSw** | **Significant Covariates** |
| **Class** | **species** | **median** | **median** | **median** | **p-value** | **p-value** | **p-value** | **p-value** |
| **LYPC** |  | 198.7 | 205.6 | 189.9 | 0.474 |  | 0.607 |  |  |
| **PC** |  | 1472 | 1399.8 | 1348.9 | 0.77 |  | 0.636 |  |  |
| **PE** |  | 140.9 | 153.6 | 142 | 0.88 | 0.485 | 0.65 | 0.581 | Age, Sex×Age, Race×Age, Race×Sex×Age |
| **LYPC** | **18:2n6** | 28.8 | 35.75 | 37.2 | 0.084 | 0.354 | 0.043 | 0.742 | Age, Sex, BMI, Race×Sex |
| **LYPC** | **18:3n6** | 0.2 | 0.2 | 0.2 | 0.343 | NA | 0.823 | NA |  |
| **LYPC** | **20:2n6** | 0.4 | 0.4 | 0.4 | 0.834 | 0.221 | 0.436 | 0.670 | BMI |
| **LYPC** | **20:3n6** | 1.8 | 1.7 | 1.95 | 0.495 | 0.028 | 0.427 | 0.815 | Race×Sex |
| **LYPC** | **20:4n6** | 8 | 9.2 | 9.85 | 0.339 | 0.227 | 0.023 | 0.230 | Age, Sex, BMI, Race×Age, BMI×Race |
| **LYPC** | **22:4n6** | 0.2 | 0.2 | 0.3 | 0.373 | 0.516 | 0.022 | 0.262 | Sex, BMI |
| **LYPC** | **22:5n6** | 0.2 | 0.2 | 0.2 | 0.720 | NA | 0.941 | NA |  |
| **LYPC** | **18:3n3** | 0.5 | 0.4 | 0.45 | 0.951 | NA | 0.758 | NA |  |
| **LYPC** | **20:5n3** | 0.4 | 0.3 | 0.4 | 0.022 | 0.040 | 0.845 | 0.514 | Sex×Age |
| **LYPC** | **22:5n3** | 0.4 | 0.4 | 0.5 | 0.355 | 0.478 | 0.092 | 0.757 | Sex, BMI, Race×Sex, BMI×Race |
| **LYPC** | **22:6n3** | 1.7 | 1.45 | 1.9 | 0.081 | 0.003* | 0.354 | 0.991 | BMI |
| **LYPC** | **PUFA** | 46 | 51.1 | 53.7 | 0.163 | 0.281 | 0.046 | 0.49 | Age, Sex, BMI, Race×Sex |
| **LYPC** | **n3** | 3.1 | 2.95 | 3.3 | 0.226 | 0.042 | 0.791 | 0.576 | BMI |
| **LYPC** | **n6** | 41.5 | 48.1 | 49.9 | 0.099 | 0.436 | 0.029 | 0.615 | Age, Sex, BMI, Race×Sex, BMI×Race |
| **PC** | **18:2n6** | 746.4 | 641.15 | 622.4 | 0.012 | 0.510 | 0.057 | 0.815 | Age, Sex, Race×Age |
| **PC** | **18:3n6** | 2.6 | 1.75 | 2.15 | 0.019 | 0.371 | 0.410 | 0.477 | Age, BMI, Sex×Age |
| **PC** | **20:2n6** | 12.2 | 8.9 | 9.55 | 0.000 | 0.183 | 0.012 | 0.093 | Age, Sex, Race×Sex, Race×Age, BMI×Sex, BMI×Sex×Race |
| **PC** | **20:3n6** | 87.6 | 62.1 | 75.5 | 0.006 | 0.652 | 0.018 | 0.096 | Age, BMI, Sex×Age, Race×Age, Race×Sex×Age, BMI×Race |
| **PC** | **20:4n6** | 397 | 320.05 | 340.7 | 0.003 | 0.193 | 0.029 | 0.361 | Age, Race, Sex, Race×Sex, BMI×Sex×Race |
| **PC** | **22:4n6** | 13.1 | 11.7 | 12.45 | 0.170 | 0.758 | 0.483 | 0.420 | Race, Race×Sex, BMI×Race, BMI×Sex×Race |
| **PC** | **22:5n6** | 10 | 9.85 | 9.5 | 0.692 | 0.519 | 0.255 | 0.692 | Sex, Race×Sex, BMI×Sex×Race |
| **PC** | **18:3n3** | 6.6 | 4.2 | 5.15 | 0.001 | 0.013^ | 0.037 | 0.066 | Age, Race×Age |
| **PC** | **20:4n3** | 2.3 | 1.15 | 1.45 | 0.001 | 0.053 | 0.026 | 0.03 | Age, BMI, Sex×Age, Race×Age, Race×Sex×Age, BMI×Race |
| **PC** | **20:5n3** | 16.3 | 7.55 | 11.45 | 0.000 | <0.001* | 0.038 | 0.046 | Age |
| **PC** | **22:5n3** | 24.9 | 19.2 | 21.6 | 0.002* | NA | 0.2 | NA |  |
| **PC** | **22:6n3** | 101.3 | 62 | 87.5 | 0.000 | 0.016^ | 0.147 | 0.656 | Age, Sex, Race×Sex, Race×Age |
| **PC** | **n3** | 155.6 | 94.8 | 122.45 | 0.000 | 0.005* | 0.077 | 0.447 | Age, Sex, Race×Sex, Race×Age, BMI×Sex×Race |
| **PC** | **n6** | 1293.5 | 1051.1 | 1095.9 | 0.001 | 0.185 | 0.021 | 0.503 | Age, Sex, Race×Sex, Race×Age, BMI×Sex×Race |
| **PE** | **18:2n6** | 29.1 | 22.15 | 26.45 | 0.003 | 0.248 | 0.095 | 0.764 | Age, Sex, Race×Sex, Race×Age, BMI×Sex |
| **PE** | **18:3n6** | 0.2 | 0.1 | 0.1 | 0.132 | 0.900 | 0.641 | 0.812 | Age |
| **PE** | **20:2n6** | 0.7 | 0.6 | 0.7 | 0.074 | NA | 0.456 | NA |  |
| **PE** | **20:3n6** | 5.1 | 3.7 | 4.7 | 0.015 | 0.233 | 0.070 | 0.253 | Age, Race, Sex×Age, Race×Age, Race×Sex×Age, BMI×Race |
| **PE** | **20:4n6** | 74.6 | 55.4 | 64.8 | 0.001 | 0.064 | 0.130 | 0.751 | Age, Race, Sex, Race×Sex, BMI×Sex×Race |
| **PE** | **22:4n6** | 2.5 | 2.5 | 2.4 | 0.737 | 0.792 | 0.830 | 0.912 | Race, BMI×Race, BMI×Sex×Race |
| **PE** | **22:5n6** | 1.6 | 1.5 | 1.8 | 0.397 | 0.992 | 0.862 | 0.237 | Race×Sex, BMI×Sex, BMI×Sex×Race |
| **PE** | **18:3n3** | 0.50 | 0.55 | 0.50 | 0.622 | NA | 0.758 | NA |  |
| **PE** | **18:4n3** | 0.10 | 0.10 | 0.10 | 0.840 | NA | 0.766 | NA |  |
| **PE** | **20:4n3** | 0.10 | 0.10 | 0.10 | 0.331 | NA | 0.351 | NA |  |
| **PE** | **20:5n3** | 1.40 | 0.90 | 1.25 | 0.001* | NA | 0.188 | NA |  |
| **PE** | **22:5n3** | 4.50 | 3.05 | 4.05 | 0.001 | 0.001* | 0.124 | 0.304 | Race×Sex, BMI×Sex×Race |
| **PE** | **22:6n3** | 18.60 | 9.90 | 16.85 | 0.000 | 0.003* | 0.163 | 0.895 | Age, Sex, Race×Sex, BMI×Sex, BMI×Sex×Race |
| **PE** | **n3** | 25 | 15.5 | 23.4 | 0.000 | 0.001* | 0.113 | 0.750 | Age, Sex, Race×Sex, BMI×Sex×Race |
| **PE** | **n6** | 116 | 86.3 | 99.1 | 0.00037^ | 0.07 | 0.09 | 0.827 | Age, Sex, Race×Sex, Race×Age, BMI×Sex×Race |

Median values represent serum concentrations (nmol/g of plasma). HC: healthy controls; FE, RE: baseline (pretreatment) values for patients with first episode or recurrent schizophrenia, respectively. LYPC: lyso-phosphatidylcholine; PC: phosphatidylcholine; PE: phosphatidylethanolamine; PUFA: polyunsaturated fatty acids. BMI: body mass index; × indicates an interaction between covariates. Paired group comparisons (Wilc: Wilcoxon test; McSw: McSweeney-Porter test) were applied to compare all lipids or fatty acids with < 33% zero concentrations. When the lipid concentration was dependent upon significant covariates, a nonparametric ANCOVA (McSweeney-Porter) was the preferred test for assessing the difference between two groups. Bonferroni alpha corrections for n3 fatty acids were: * p<.05/7=0.0071 for PE ; p<0.05/6=0.0083 for PC, and p<0.05/5=0.01 for LYPC; ^ p<0.10/7=0.014 for PE, p<0.10/6=0.017 for PC, and p<0.1/5=0.02 for LYPC. Bonferroni alpha corrections for n6 fatty acids were: †p<.05/8=0.00625; ‡ p<0.1/8=0.0125.
